# Supplementary figures and images for: The distribution and numbers of cheetah (Acinonyx jubatus) in southern Africa
Source: PeerJ. 2017 Dec 11;5:e4096. doi: 10.7717/peerj.4096 (PMC5729830; doi:10.7717/peerj.4096)

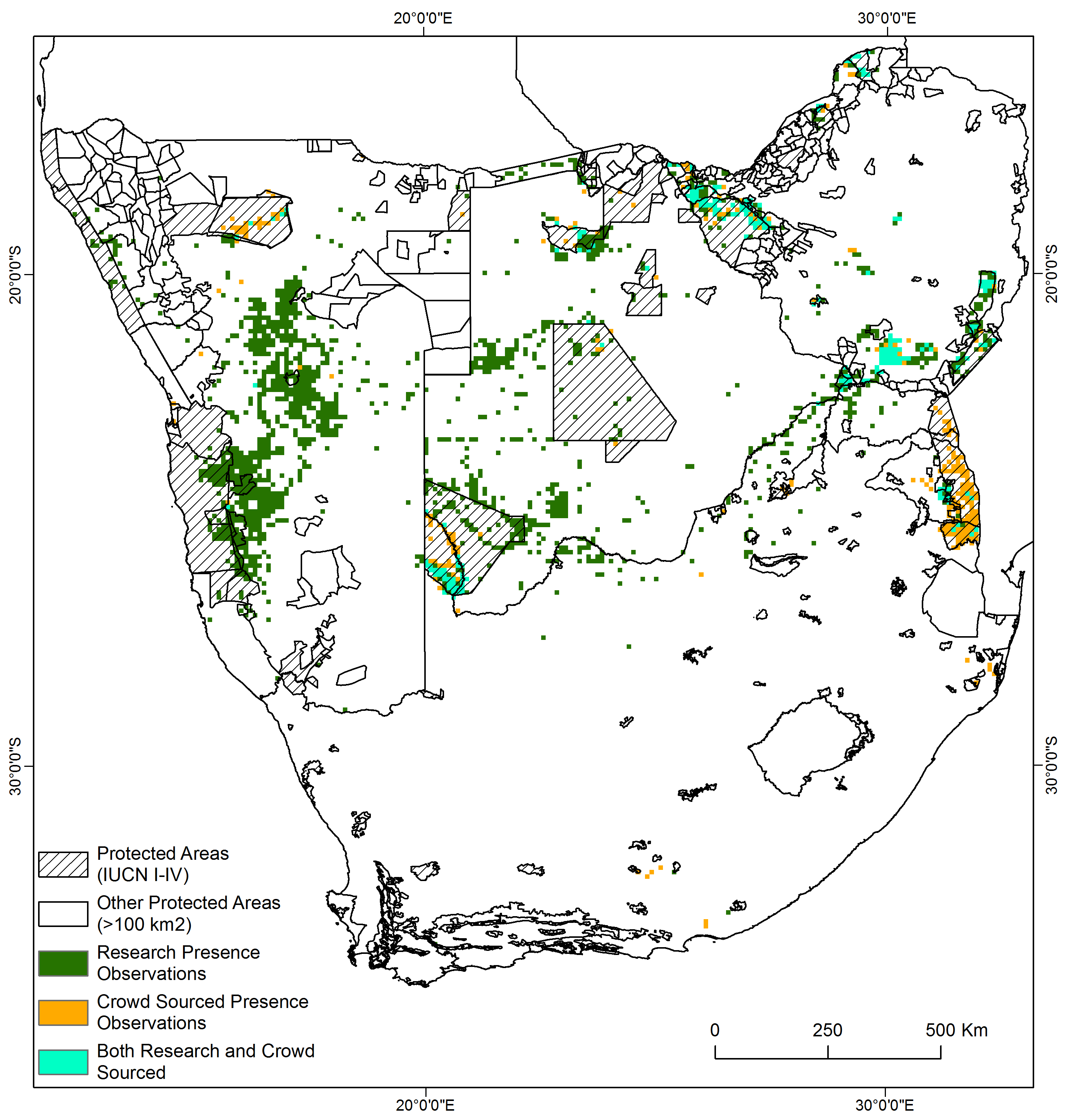

Supplement: Figure S1 [file peerj-05-4096-s001.png]

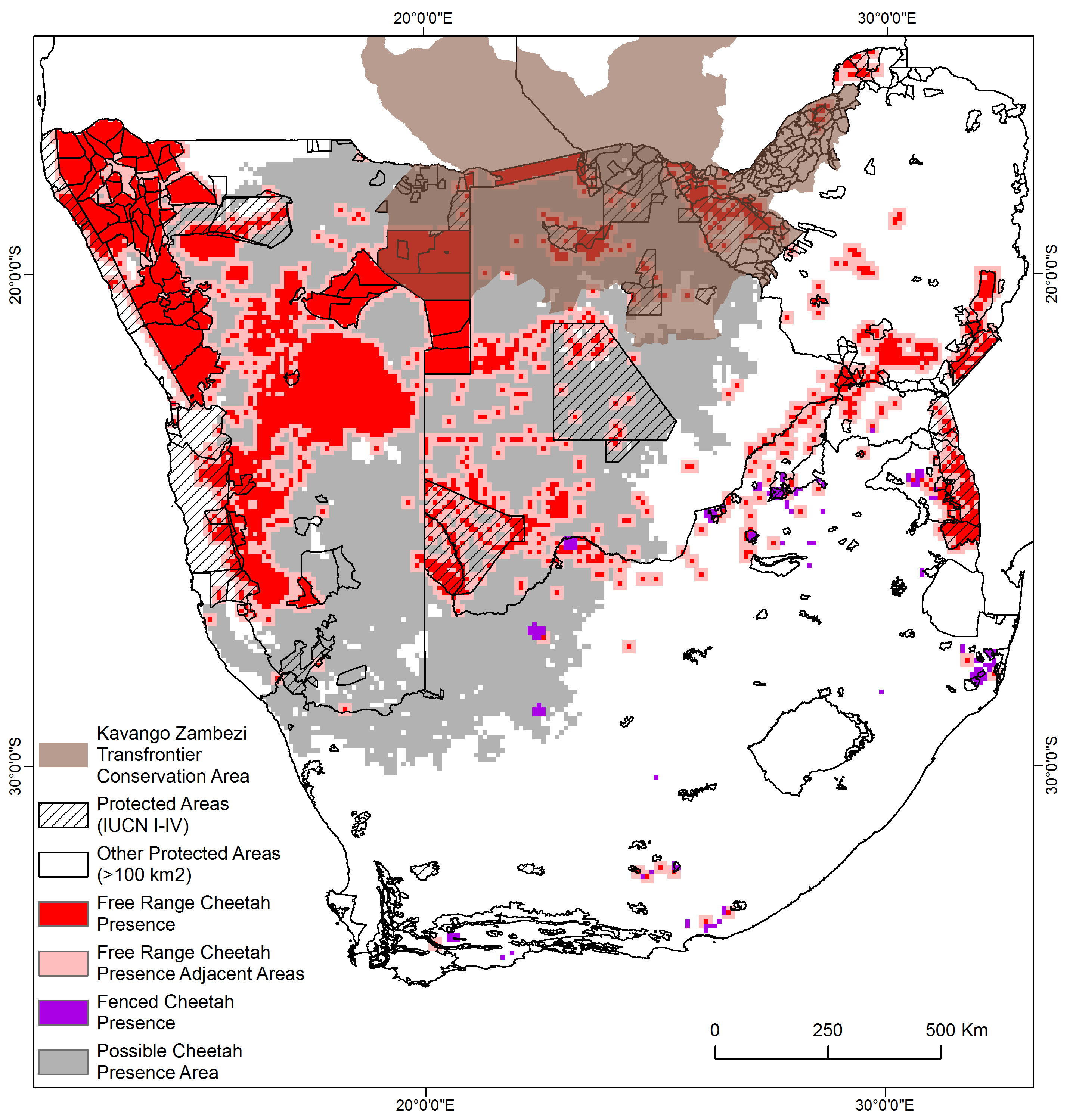

Supplement: Figure S2 [file peerj-05-4096-s002.png]

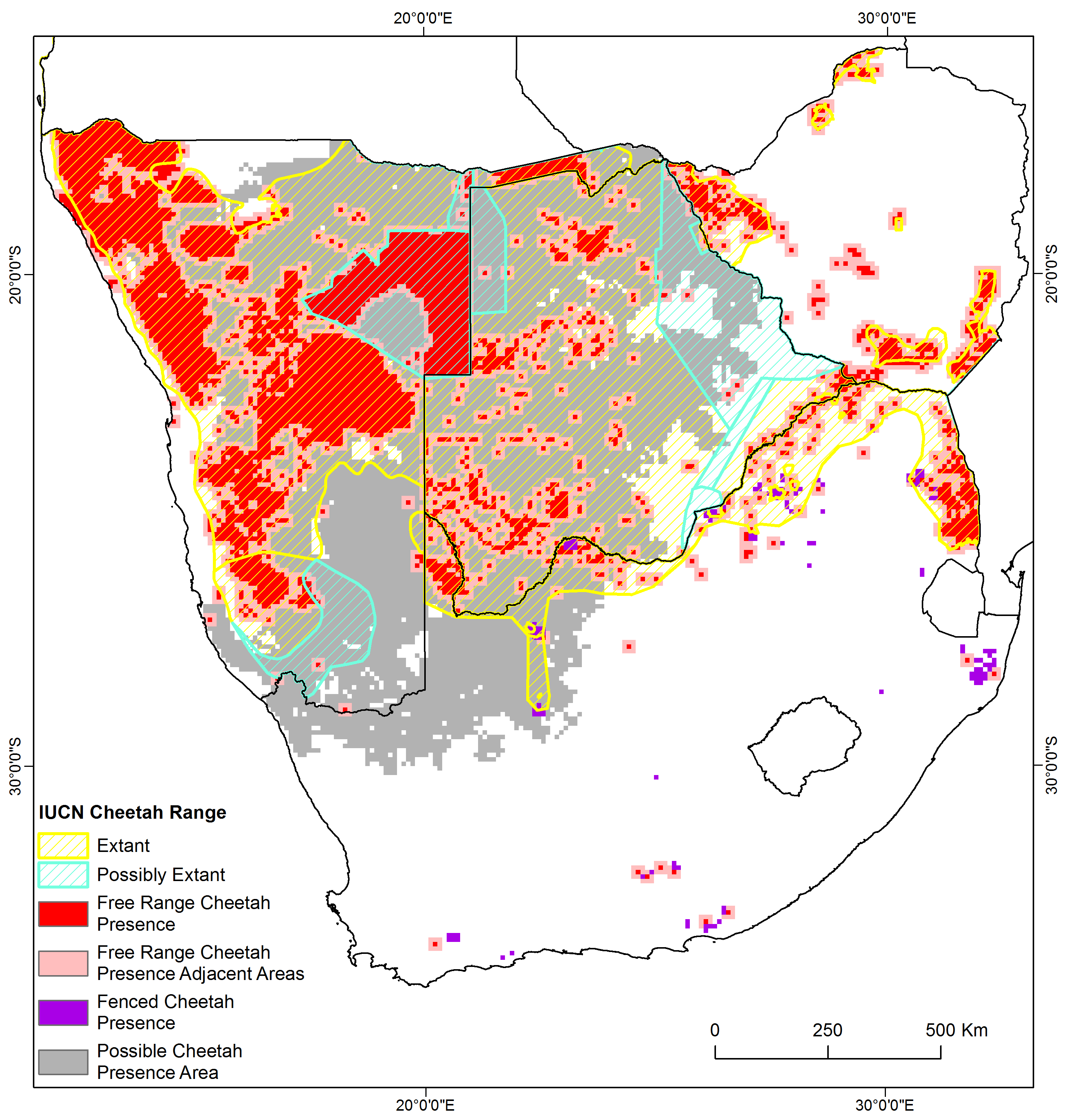

Supplement: Figure S3 [file peerj-05-4096-s003.png]
