# Supplementary material for: The distribution and numbers of cheetah (Acinonyx jubatus) in southern Africa
Source: PeerJ. 2017 Dec 11;5:e4096. doi: 10.7717/peerj.4096 (PMC5729830; doi:10.7717/peerj.4096)
Supplement: Appendix 3 [file peerj-05-4096-s006.doc]

**Appendix 2**

*Presence data refining*

We discarded internet records and other non-research data without supporting images or video, dates, or which lacked accurate locations. We eliminated obvious false entries (e.g. leopard records labelled as cheetah observations) and duplicate records of the same observation. Prior to final analyses, we reviewed databases critically to check the accuracy of the information and to interrogate unusual records. Where available, we sourced exact latitude and longitude information from image meta-data. For public records not giving exact location detail, we georeferenced them using the nearest verifiable location mentioned in the description of the records (Table 1). For example, records including a specific farm, lodge, waterhole, road junction, or other distinct geographic features were georeferenced to that location. Records only providing coarse geographic reference (e.g. “cheetah in Kruger NP”) without further detail were discarded. This, and the fact that records from some public databases are automatically obscured to protect the species, means that mapping data incurred some degree of location error. This error does not exceed 25 km for any of the data we used in our analyses. It is likely negligible for mapping the distribution of this highly mobile species that can range over > 500km^2^ (Caro 1994). Also, we only considered records with a reliable time reference. Except for the managed cheetah population in South Africa, we excluded records relating to cheetahs maintained in effective confinement, for example at wildlife rehabilitation centres or on tourism farms. Within communal conservancies in Namibia, data is only presence/absence for the entire conservancy and does not link to an exact GPS coordinate. Finally, for the fenced meta-population of South Africa, we did not have the exact boundaries of the properties. Instead, we had a centroid and the area of the fenced population, therefore we approximated the extent as a circle with area matching the given area.

To reduce auto-correlation in telemetry data, we pruned available GPS and VHF locations to a single location per animal or cohesive social group per day, closest to 12:00 local time. Also, we pruned telemetry data to eliminate immediate post-translocation explorations of relocated individuals and thus only settled movements were considered for mapping and analyses (following Weise et al. 2015).

The exact location data are stored on Dryad (**DOI XXX**). If so requested by contributors, we removed property and land ownership details from publishable data to protect identities. Similarly, we obscured original latitude-longitude information of sensitive research records; these may be sourced from the contacts provided.

Table 1. Summary of presence data by country and split by source, and if the exact GPS point was given.

| Country | No. presence data (% of total) | Non-research | Research | Exact GPS | Nearest GPS |
| --- | --- | --- | --- | --- | --- |
| Botswana | 4,172 (21%) | 145 | 4,027 | 3,930 | 242 |
| Namibia | 10,226 (51%) | 578 | 9,648 | 9,642 | 584 |
| South Africa | 3,514 (17%) | 2,321 | 1,193 | 2,169 | 1,345 |
| Zimbabwe | 2,202 (11%) | 564 | 1,638 | 2,201 | 1 |
| Total | **20,114** | 3,608 | 16,506 | 17,942 | 2,172 |
| Percentage (total) | **100** | **17.9** | **82.1** | **89.2** | **10.8** |

References

Caro, T. 2004. Cheetahs of the Serengeti Plains. University of Chicago Press, Chicago.

DWNP. 2013. Division of Wildlife and National Parks, Botswana. Aerial census of animals in Botswana. Research and Statistics Division, Gaborone.

Weise FJ, Lemeris Jr J, Munro S, Bowden A, Venter C, van Vuuren M, van Vuuren R. 2015. Cheetahs (*Acinonyx jubatus*) running the gauntlet: an evaluation of translocations into free-range environments in Namibia. PeerJ (e1346) DOI: 10.7717/peerj.1346.

Weise FJ. 2016. An evaluation of large carnivore translocations into free-range environments in Namibia. Doctoral thesis (PhD), Manchester Metropolitan University.

RWCP, IUCN/SSC. 2015. Regional conservation strategy for the cheetah and African wild dog in southern Africa; revised and updated, August 2015.

Van der Meer E. 2016. The cheetahs of Zimbabwe, distribution and population status 2015. Cheetah Conservation Project Zimbabwe, Victoria Falls, Zimbabwe. DOI: 10.13140/RG.2.2.36719.84648
